# Methodology to study polymers interaction by surface plasmon resonance imaging^☆^

**DOI:** 10.1016/j.mex.2014.12.001

**Published:** 2014-12-15

**Authors:** N. Vollmer, F. Trombini, M. Hely, S. Bellon, K. Mercier, C. Cazeneuve

**Affiliations:** a HORIBA Scientific, Avenue de la Vauve, Passage Jobin Yvon, 91120 Palaiseau Cedex, France; b L'Oréal Research and Innovation, 1 Avenue Eugène Schueller, 93601 Aulnay Sous Bois, France

**Keywords:** Detection of polymers interaction, Surface plasmon resonance imaging, Interactions, Kinetic profile, Association phase, Dissociation phase, Polymers, Reversible inclusion complex

## Abstract

The surface plasmon resonance (SPR) technique has been primarily used in the field of biology, in particular for the study of antibody-antigen interactions. Recently, polymers were introduced to form inclusion complexes.

We describe here, a methodology based on surface plasmon resonance imaging to study water-resistant and reversible inclusion complexes using systems which are compatible with a cosmetic use. The purpose of this study is to follow in real time the interaction between two polymers.

To carry out this study:

• A biochip based on a covalent binding of one “host polymer” on a gold-activated surface was developed.
• The binding of the host polymer to a guest polymer was monitored.
• The presence of interactions between the β-cyclodextrins groups of the host polymer and the adamantyl functional groups of the guest polymer and the possibility of dissociating the complex were established.

This technique allowed carrying out parallel assays for optimizing the amount of complexes formed, the host polymer being spotted at five concentrations. It was then possible to study the influence of the concentration in host system for two concentrations of the guest polymer. The concentration in the host polymer yielding the highest immobilization of the guest system was further determined.

## Graphical abstract

**Figure:**
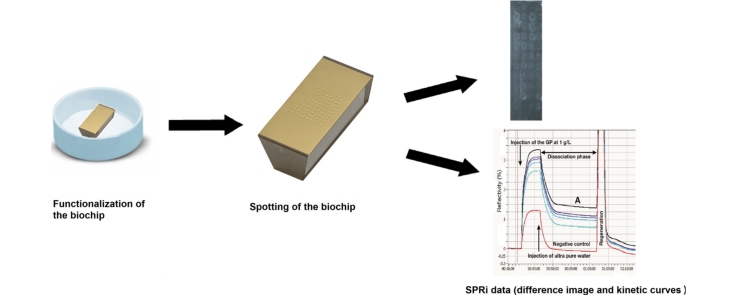

## Method details

### Methodology to study polymers interaction by surface plasmon resonance imaging

Usually surface plasmon resonance imaging (SPRi) is used in biology to characterize interactions between two biological molecules, but in this article we propose a methodology to follow interactions between two polymers. In this study, a host polymer (HP) is immobilized on the surface of a biochip and a guest polymer (GP) is injected on the SPRi-Biochip™ in order to monitor these interactions by SPRi.

Owing to this technique, the association and the dissociation of both polymers can be observed in real-time on kinetic curves (increasing or decreasing reflectivity variation, respectively) and on the SPRi difference images where spots become white during the association phase and turn black during the dissociation phase. The guest polymer could be dissociated from the host polymer by using a surfactant solution.

The final goal of this work is to create new structures and new materials. In inclusion complexes, one of the compounds, the system host, forms a cavity in which a second molecule is located. The systems find applications for the development of multilayered structures that can be used as reversible active ingredient matrices.

### Host and guest polymers

1) The host polymer (HP) was obtained from polycondensation between ß-cyclodextrins and epichlorohydrin [1]. It was amine functionalized for binding with carboxylic acid groups on the gold layer of the SPRi chip. This polymer had a molecular weight of 30 kDa and contained on average 20 ß-cyclodextrin groups per chain and 60 amine functions distributed along the chain. The conditions for the synthesis and the analytical characterizations of this polymer are described elsewhere [2].
2) The guest polymer (GP) was a four arm star poly(ethylene glycol) (PEG) with a molecular weight of 20 kDa. Each arm had an adamantyl function at its end. This adamantyl modified poly(ethylene glycol) was prepared according to the process described in a previous work [3] from adamantyl carbonyl chloride and “4 Arm PEG Amine, 20 kDa”, supplied by Sigma–Aldrich and Nanocs, respectively.
3) A sodium dodecyl sulfate (SDS), regeneration solution, was used at a concentration of 10 g/L in ultrapure water, above the critical micelle concentration (2.7 g/L) to be sure that the desorption of the guest polymer occurs.

**Fig. 1:**
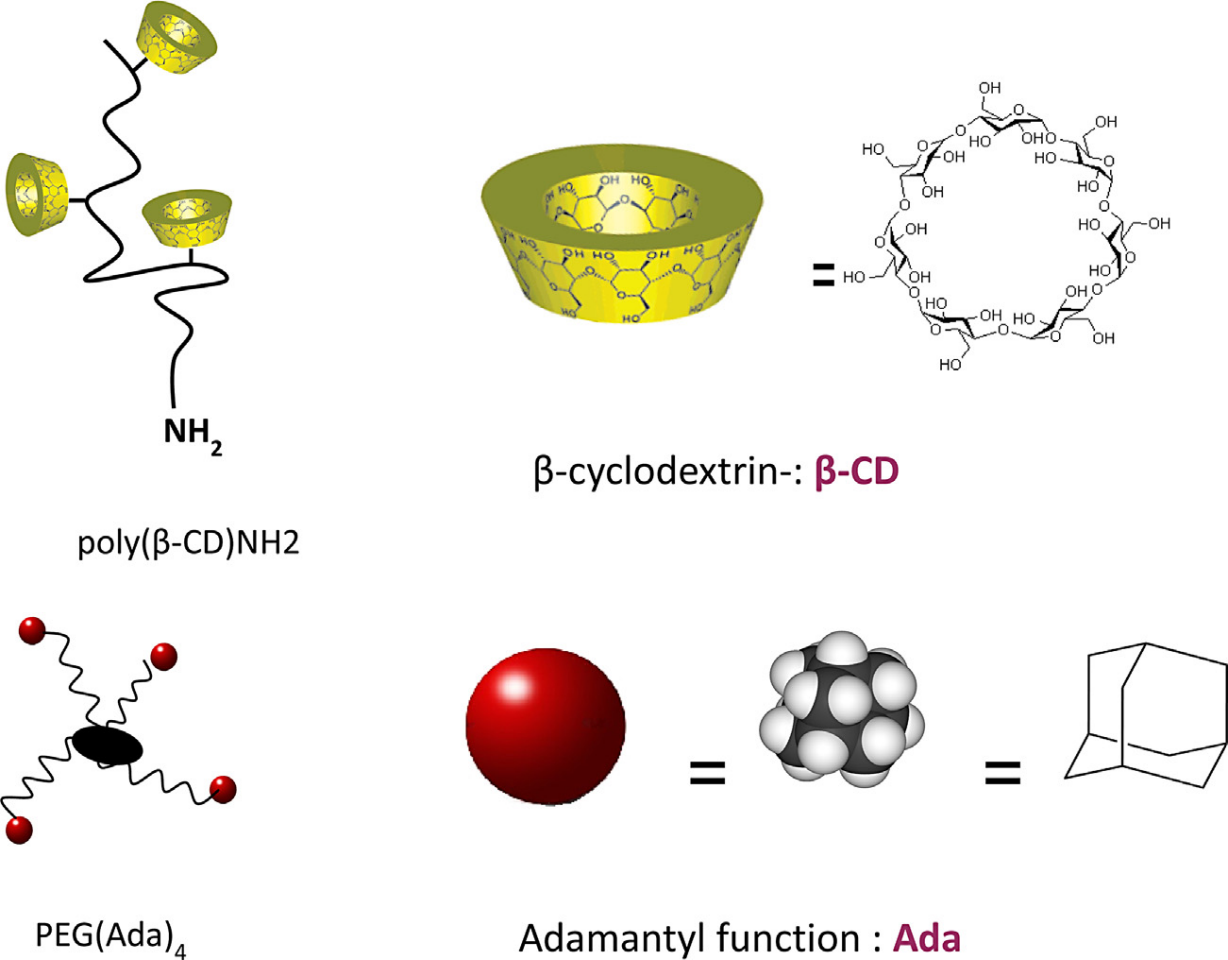
Schematics of the chemical structure of the host polymer poly(β-CD)NH2 and the guest polymer PEG(Ada)_4_.

Fig. 1 represents the schematics of the chemical structure of the host and guest polymers.

### Immobilization of the host polymer on the SPRi-Biochip™

**Fig. 2:**
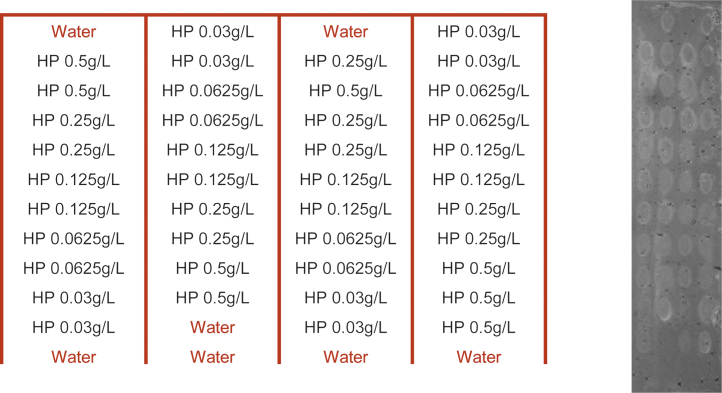
Image and spotting map of the spotted host polymer (HP).

1) The host polymer (HP), used as a receptor, was immobilized covalently on a CS-LD SPRi-Biochip™ provided by HORIBA Scientific. The CS-LD SPRi-Biochip is a low density biochip recommended to immobilize big molecules such as polymers. The surface chemistry of this SPRi-Biochips™ is based on amine coupling using NHS groups [4]. The CS SPRi-Biochip is stored under argon because it is sensitive to humidity and in the dark. As a result, the biochip is unpacked just before the immobilization the host polymer. The host polymer was immobilized at five different concentrations (0.03, 0.06, 0.12, 0.25 and 0.50 g/L) directly on the functionalized biochip without cross linker. The HP was diluted in ultrapure water (milliQ water).
2) Each concentration was spotted 8 or 9 times, to produce an array of HP active sites with differing concentrations for a total of 48 spots. The spots marked as water were considered as negative controls and corresponded to areas spotted with ultrapure water.
3) Half of a low 96-wells microplate supplied by VWR was filled with 70 μL of HP solution or water in each well in order to perform a 12 × 4 matrix as indicated in Fig. 2.
4) The microplate was then inserted in the SPRi-Continuous Flow Microspotter™ (SPRi-CFM™) supplied by HORIBA Scientific to carry out the spotting of the samples. The SPRi-CFM™ printer, using a flow deposition, allows increasing the spots homogeneity and guaranteeing a better density of immobilized molecules. A printhead linked to 48 needles that pick up the samples in the microplate, goes in contact with the region of interest of the biochip and each sample solution goes back and forth (cycle of 1 min) for 15 min at 60 μL/min. The attachment of the host polymer is carried out via amino groups of the host polymer to the *N*-hydroxy succinimide groups of the modified gold surface.
5) After the printing procedure, the biochip was removed from the SPRi-CFM and rinsed with distilled water without any drying.
6) The free *N*-hydroxy succinimide groups on the gold surface that did not react with the host polymer, were blocked for 15 min by immersing the biochip in a 0.5 mol/L pH 7.5 ethanolamine solution under agitation to improve the contact between ethanolamine and the biochip surface in order to optimize the blocking and in the dark to avoid the damage of the gold-SH link used in the first step of the biochip functionalization.
7) After this blocking step, the biochip was rinsed with ultrapure water and dried with a gentle air flow.

### Monitoring the interaction between the host and guest polymers

1) First the SPRi system was filled at high flow rate (500 μL/min) with the running buffer (ultrapure water).
2) Then the spotted SPRi-Biochip™ was inserted into the analysis chamber of the SPRi-Plex II™ instrument (HORIBA Scientific). The flow rate was fixed at 50 μL/min and the experiment was carried out at 25 °C.
3) Each region of interest (HP spots and water spots) was selected and identified.
4) The SPRi system allows plotting plasmon curves (the reflectivity variation versus incidence angle) for each spot in order to monitor the reflectivity variation at a fixed incidence angle chosen where the plasmon slope is globally higher for each spot.
5) Two solutions of the guest polymer (GP) diluted in ultrapure water at 1.0 g/L and 0.1 g/L were each sequentially injected in the flow cell for 5 min. Between each GP injection, the chip was rinsed with ultrapure water to remove unbound guest polymer for around 15 min.
6) The interaction between the two polymers (host and guest) was monitored in real time by SPR imaging. The reflectivity variations of the chip active spotted matrix and hence the host polymer/guest polymer interactions were measured simultaneously for the complete chip matrix.

### Formation of inclusion complexes

**Fig. 3:**
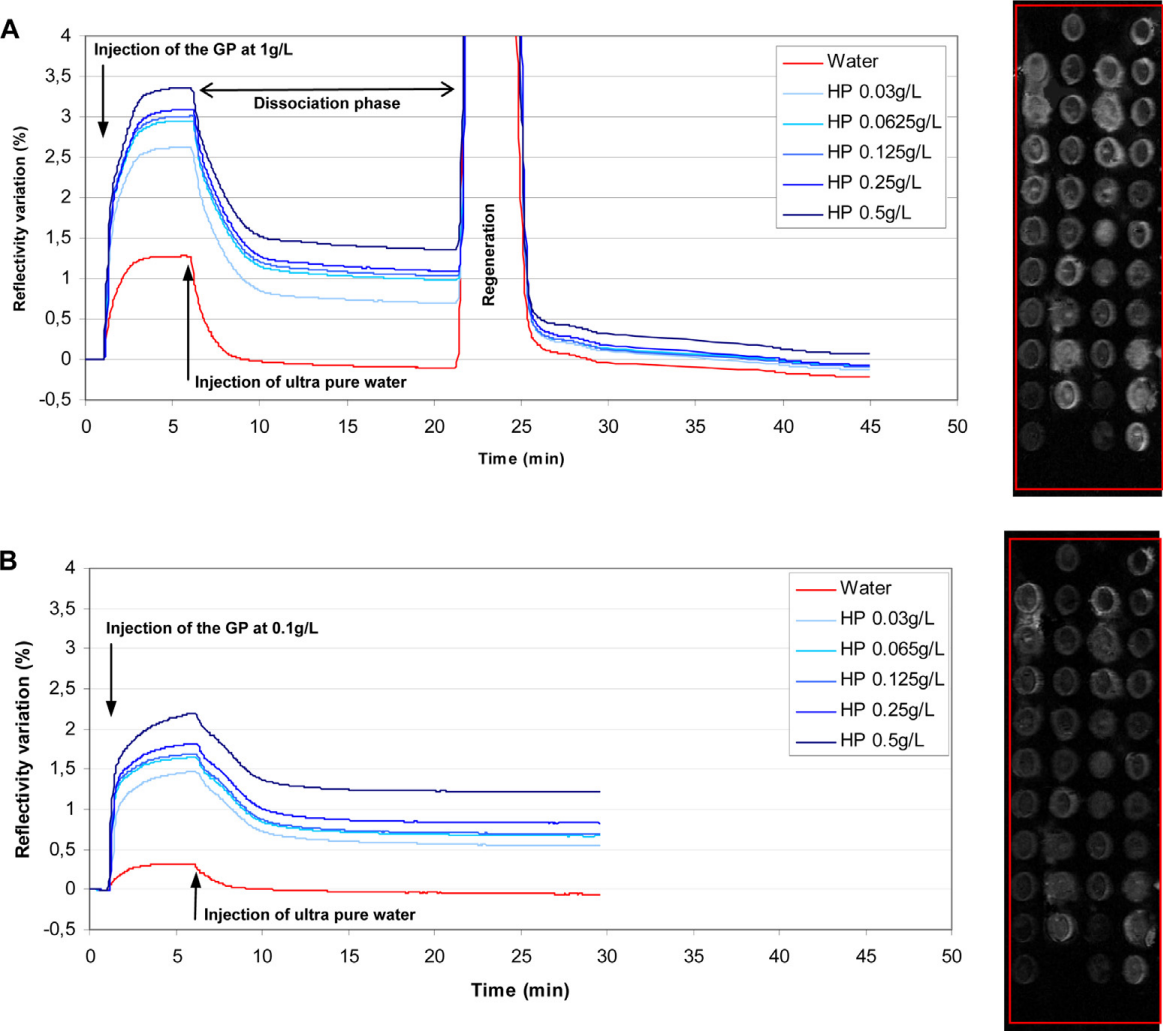
Kinetic curves and difference images after injecting the guest polymer at (A) 1.0g/L and (B) 0.1g/L. The blue curves correspond to HP/GP interactions and the red curve corresponds to the SPRi response on negative control spots. Active sites, where interaction has occurred, show up as bright spots and dark areas showing no active interaction, correspond to the control “water” sites (see map Fig. 2).

The kinetic curves (Fig. 3) show the association/dissociation profiles between the host polymer immobilized on the surface of the biochip and the guest polymer injected at 1 g/L and 0.1 g/L. Each kinetic curve corresponds to an average curve of the 8 or 9 spots used for each concentration, providing a robust and accurate average value.

As shown in Fig. 3, a specific response is observed on the host polymer spots with no variation of reflectivity on “water” spots. In fact, when considering the signal before injecting the guest polymer and after injecting the circulating fluid, the signal returns to baseline. The variation in the reflectivity on “water” spots during the injection of the guest polymer results from the difference of refractive index between the highly concentrated polymer solution injected and the circulating fluid (ultrapure water). In addition, the intensity of SPRi response increases with increasing concentrations of the spotted host polymer.

These interactions are specific. PEG derivatives without the adamantyl end group have been tested elsewhere [2], there is no adsorption of unmodified PEGs onto β-CD polymer coated gold surface.

In the absence of the host polymer no capture of the guest polymer is recorded, demonstrating that the species adsorbed on the spots of the host polymer are obtained via specific interactions between adamantyl groups and ß-CD cavities. These inclusion complexes are formed quickly. We detect the interaction between the two polymers within several minutes. The interactions between adamantyl groups and ß-CD cavities are irreversible in water, i.e., the curves do not return to baseline. These complexes are water resistant.

The changes in the SPRi response on the polymer spots can be followed on both the kinetic curves and the real-time images (flow cell images and difference images) provided by the camera. The difference image of the biochip surface is obtained by subtracting a reference image (taken just before the guest polymer injection) from each image taken during the measurement. In this way we can observe directly the binding of the guest polymer on the difference image in real time. The spots where the interaction occurs become white whereas the spots where there is no interaction remain black.

The polymer spots where interaction occurred are shown as bright active spots whereas the negative control spots remain dark. In order to determine the influence of the concentration of the polymer with adamantyl groups (GP), a 0.1 g/L solution was passed over the β-CD polymer (HP). The active HP spots are less intense after injecting the guest polymer at 0.1 g/L than at 1 g/L. This result confirms that SPRi response also depends upon the injected concentration.

### Dissociation of the complexes – regeneration of the interaction

The interaction was regenerated by injecting a concentrated solution of surfactants, sodium dodecyl-sulfate (SDS 1%) for 2 min. The regeneration step consists in disrupting the interaction between guest and host polymers. A desorption profile of the guest polymer was observed in this process, whilst the host polymer remained immobilized and unchanged (Fig. 3). The surfactant solution was a suitable regeneration solution since it enabled the starting conditions to be recovered after regeneration. The use of a regenerative step allows the experimental measurements to be repeated in consecutive analyses (or altered conditions if necessary) without requiring and preparing any further chips. This improves the speed of analysis and reduces the number of biochips used. The sodium dodecyl-sulfate is known to form inclusion complexes with ß-CD cavities [5], [6]. There is nearly a complete desorption of immobilized adamantyl modified PEG layer. Indeed the interaction between the HP and adamantyl is largely based on hydrophobic interaction, that is why the surfactant is effective to remove the guest polymer.

These experiments demonstrate that the SPR imaging platform is well adapted to the characterization of polymer/polymer interactions and enable a quicker and easier determination of the optimal experimental conditions due to the multiplexed chip format.

Having covalently bound the host polymer on a carboxylic acid-modified gold surface, the capture of the guest polymer, a star poly(ethylene glycol) with four adamantyl groups, occurs in mild conditions (aqueous environment, room temperature) and with fast kinetics. It was shown that the immobilization was achieved through the creation of inclusion complexes. The immobilized complexes have the advantage of being easily abolished through exposure to a surfactant solution. The conditions yielding the optimal amount of inclusion complexes and thus the highest adsorption of the guest polymer are found with concentrations of 0.5 g/L and 1 g/L of host and guest polymer solutions, respectively.
