# Supplementary material for: Methodology to study polymers interaction by surface plasmon resonance imaging
Source: MethodsX. 2014 Dec 15;2:14–8. doi: 10.1016/j.mex.2014.12.001 (PMC4487328; doi:10.1016/j.mex.2014.12.001)
Supplement: Supplementary file 1 [file mmc1.docx]

**Additional Information**

Surface Plasmon Resonance (SPR) is an optical phenomenon that occurs when a polarized light beam hits a prism covered by a thin gold layer. Under certain conditions (wavelength, polarization and incidence angle) free electrons at the surface of the SPRi-biochip™ absorb the incident light photons and convert them into surface plasmon waves. At a given angle, called resonance angle, the intensity of reflected light is minimal. Perturbations at the gold/solution interface, such as interactions between probe molecules immobilized on the biochip and captured target molecules, alter the conditions of resonance.

Surface Plasmon Resonance imaging (SPRi) is a sensitive label-free method for visualizing the region of interest of the biochip via a CCD video camera. The high resolution CCD video camera provides real-time difference images across an array of immobilized molecule. This design allows us to use a pattern with different immobilized molecules and simultaneously follow up the kinetic curves of each spotted molecule. In this SPRi configuration, the measured local changes in light reflectivity are proportional to the amount of target molecules captured each spot. The local changes at the surface of the chip provide detailed information on molecular binding and kinetic processes.
